# Development of active jejunal glucose absorption in broiler chickens

**DOI:** 10.1016/j.psj.2023.102804

**Published:** 2023-05-23

**Authors:** Mikako Shibata, Tatsuyuki Takahashi, Takaharu Kozakai, Junji Shindo, Yohei Kurose

**Affiliations:** ⁎ Laboratory of Animal Metabolism and Function, School of Veterinary Medicine, Kitasato University, Towada, Aomori, Japan; † Faculty of Education, Art and Science, Yamagata University, Yamagata, Japan; ‡ Laboratory of Wildlife Science, School of Veterinary Medicine, Kitasato University, Towada, Aomori, Japan

**Keywords:** jejunal glucose absorption, jejunal permeability, jejunal morphology, age, broiler

## Abstract

Growth in chickens, especially meat-type chickens (broilers), is extremely rapid, but studies on the regulatory mechanism of intestinal glucose absorption with growth are few, contradictory, and unclear. Here, we investigated the regulation of intestinal glucose absorption with growth in broiler chickens using oral glucose gavage, intestinal Evans blue transit, intestinal glucose absorption, scanning electron microscopy, and glucose absorption- and cell junction-related gene expression analyses. Peak blood glucose levels after oral glucose gavage occurred at 10 and 50 min in chickens at 1 wk (**C1W**) and 5 wk (**C5W**) of age, respectively. The area under the curve for glucose levels was greater for the C5W than the C1W (*P* = 0.035). The stain ratio in the small intestine in the C5W was lower than that in the C1W (*P* = 0.01), but there were no differences in the tissue regions stained with Evans blue and the migration distance of Evans blue from Meckel's diverticulum. In everted sac and Ussing chamber experiments, we observed reduced intestinal glucose uptake and electrogenic glucose absorption in the jejunum of the C5W. Phloridzin, an inhibitor of sodium/glucose cotransporter 1 (**SGLT1**), suppressed the glucose-induced short-circuit current in the C1W (*P* = 0.016) but not the C5W. Although the addition of NaCl solution stimulated the glucose-induced short-circuit current in the C1W, no differences between the treatments were observed (*P* = 0.056), which was also the case in the C5W. Additionally, tissue conductance was diminished in the C5W compared with that in the C1W. Moreover, in the C5W, the intestinal tract was more developed and the jejunal villi were enlarged. In conclusion, glucose absorption throughout the intestine could be greater in C5W than in C1W; however, reduced SGLT1 sensitivity, decreased ion permeability, and intestinal overdevelopment lead to decreased local glucose absorption in the jejunum with growth in broiler chickens. These data provide a detailed analysis of intestinal glucose absorption in growing broiler chickens, and can contribute to the development of novel feeds.

## INTRODUCTION

Chickens are the most popular poultry, and they have been specialized for meat and egg production through breeding and genetic selection over a long period of time. Meat-type chickens, known as broilers, exhibit dramatic body weight gain with growth, and their body weight at slaughter (5–7-wk old) increases by more than 50-fold compared with that at hatching (Zuidhof et al., 2014). In contrast, the body weight of 5- to 7-wk-old egg-type chickens is approximately 10-fold than that at hatching (Barfull et al., 2002). Therefore, although meat- and egg-type chickens are the same species, they have completely different characteristics, abilities, and growth rates. To clarify the factors associated with rapid growth in meat-type chickens, studies have mainly discussed the mechanisms and associations of feed intake and energy metabolism in the brain, liver, adipose, and muscle tissues (Jonaidi et al., 2002; Richards, 2003; Torchon et al., 2017; Hu et al., 2019; Soglia et al., 2019). Effective nutrient absorption is one of the primary conditions for raid body growth in chickens. However, only a few studies have examined intestinal nutrient absorption and its regulation with age in broiler chickens.

Most nutrients are taken up from the small intestine via the transcellular and/or paracellular pathway (Karasov, 2017). In the mammalian transcellular pathway, intestinal glucose is primarily transported via sodium/glucose cotransporter 1 (**SGLT1**), glucose transporter 2 (**GLUT2**), and Na^+^/K^+^-ATPase (Gorboulev et al., 2012). Among these factors, many studies have focused on SGLT1 regulation because it plays a major role in intestinal glucose absorption. Although intestinal SGLT1 protein levels increase with age, glucose absorption per intestinal tissue and mucosal weight and surface area are reduced in rats (Drozdowski et al., 2003). In chickens, glucose is similarly transported into intestinal epithelial cells via the same system as mammalian carrier-mediated transportation (Riesenfeld et al., 1980). However, the SGLT1 protein level in the jejunal brush-border membrane (**BBM**) of egg-laying chickens decreases with growth without changes in mRNA expression, resulting in reduced methyl-α-D-glucopyranoside transport across the BBM (Barfull et al., 2002). Recently, we found that maltose induced an increase in the short-circuit current (*I*_sc_) for active transport indicators in the jejunum of 1-wk-old broiler chicks compared with that of 5-wk-old broiler chickens, whereas variations in maltase activity were not observed (Shibata et al., 2019). This finding supports that Na^+^-dependent intestinal absorption of glucose produced by maltose digestion varies with age in broiler chickens. However, SGLT1 mRNA expression levels gradually increase from embryo d 20 to posthatching d 14 (Gilbert et al., 2007). Additionally, Obst and Jared (1992) observed that intestinal glucose absorption increases with age in 2-, 5-, and 10-wk-old layer chickens. Thus, findings on intestinal glucose absorption in growing chickens are conflicting, and factors other than SGLT1 must be considered.

To clarify the mechanisms by which intestinal glucose absorption varies with age, it is necessary to examine both transcellular and paracellular ways. Loss of the tight junction protein claudin-15 elicits glucose malabsorption due to insufficient Na^+^ recycling from the submucosa to the lumen in the small intestine of adult mice, that is, reduced paracellular Na^+^ permeability (Tamura et al., 2011). Since intestinal glucose absorption in chickens is susceptible to luminal Na^+^ concentrations (Ferraris, 2001), paracellular Na^+^ permeability may also be involved in the age-related variation of glucose absorption in broiler chickens. This study aimed to examine age-dependent regulation of jejunal glucose absorption, tissue conductance as paracellular ion permeability, and glucose absorption- and cell junction-related gene expression in broiler chickens. We also assessed jejunal morphological characteristics.

## MATERIALS AND METHODS

### Reagents

The reagents used in this study were as follows: pentobarbital (Nacalai Tesque, Kyoto, Japan) for euthanasia, Evans blue (Kanto Chemical Co., Inc., Tokyo, Japan) for the Evans blue transit test, D-mannitol (Fujifilm Wako Pure Chemical Corporation, Osaka, Japan), phlorizin (Fujifilm Wako Pure Chemical Corporation, Osaka, Japan) for the Ussing chamber experiment, glutaraldehyde (Fujifilm Wako Pure Chemical Corporation, Osaka, Japan), and tannic acid (Fujifilm Wako Pure Chemical Corporation, Osaka, Japan) for scanning electron microscopy, N-methyl-D-glucamine (Sigma-Aldrich, St. Louis, MO) for the Ussing chamber experiment, osmium tetroxide (Sigma-Aldrich, St. Louis, MO) for scanning electron microscopy, and ouabain (R&D Systems, Minneapolis, MN) for the Ussing chamber experiment.

### Animals and Diets

All experiments carried out in this study were approved by the Institutional Animal Care and Use Committee of Kitasato University (Approval #16-064). Day-of-hatch male broiler chicks (UK chunky; *Gallus gallus domesticus*) were provided by Prifoods Co., Ltd. (Aomori, Japan) and assessed at 1- and 5-wk old. As in our previous study, the chicks were kept in brooders maintained at a temperature of 28°C ± 2°C until 3 wk after hatching and then in individual cages at 26°C ± 2°C until 5 wk of age (Shibata et al., 2019). Birds in the brooders and individual housing were kept under 24-h continuous lighting. To minimize the influence of diet on the experimental results, all roosters were offered the same diet (Chubushiryo Co., Ltd., Aichi, Japan) and given water and feed ad libitum. For all experiments, chicks at 1 wk (**C1W**) and 5 wk (**C5W**) of age were randomly selected. To empty the intestinal contents of these chicks and chickens, the birds were fasted for 12 h (C1W) and 24 h (C5W) prior to all experiments, respectively.

### Tissue Preparation

Chickens were anesthetized by pentobarbital overdose (100 mg/kg BW, i.p.) and sacrificed by exsanguination. The small intestine was immediately removed, and the intestinal length was measured from the duodenum to the distal ileum. Thereafter, the tissues were cut to differentiate each intestinal segment (duodenum, proximal and distal jejunum, and proximal and distal ileum). All intestinal regions were tested in everted sac trials, and the distal jejunum was examined in short-circuit current trials and by scanning electron microscopy and qPCR.

### Oral Glucose Administration Test

C1W (*n* = 10: 170 ± 5 g BW) and C5W (*n* = 10: 2,413 ± 62 g BW) received a glucose solution (2 g/kg BW) immediately after measuring blood glucose levels from the nail at time 0 with a Glucocard DIA meter-α (ARKRAY Co., Ltd., Kyoto, Japan). Subsequently, the glucose levels were measured at 10, 20, 30, 40, 50, 60, 90, and 120 min. Additionally, the area under the curve (**AUC**) of both groups was analyzed.

### Small Intestinal Evans Blue Transit

Small intestinal transit was determined by methods employing Evans blue as described previously (De Schepper et al., 2007; Zhang et al., 2012). The C1W (*n* = *7*; 183 ± 3 g BW) or C5W (*n* = 5; 2,450 ± 166 g BW) were provided glucose solution that included 1% Evans blue solution via gavage tube. Twenty minutes later, the birds were anesthetized and sacrificed. Subsequently, the gastrointestinal tract was immediately excised, after which the length of the entire small intestine and the distance from the beginning of the duodenum or Meckel's diverticulum (**MD**) to the most distal migration point of Evans blue were measured. The location of the MD was defined as zero. The stained proximal region from the MD was expressed as a negative value, whereas the stained distal region from it was expressed as a positive value. Transit was expressed as centimeter migration and the percentage of the small intestine stained by Evans blue.

### Everted Sac Technique

Glucose uptake across the intestine was analyzed in C1W (*n* = 7; 113 ± 7 g BW) and C5W (*n* = 5; 2,158 ± 40 g BW) using the everted gut sac method (Stelzner et al., 2001). Briefly, each intestinal segment (approximately 5 cm in length; duodenum, proximal and distal jejunum, and proximal and distal ileum) was placed into cold PBS on ice, and the mesenterium and fat were removed. Afterward, tissues were washed with cold-PBS to remove the intestinal contents and everted using a plastic rod. One end was tied using sutures, and then the sac was loaded with Krebs Ringer buffer solution (**KRB**: 140 mM NaCl, 5.0 mM KCl, 2.0 mM CaCl_2_·2H_2_O, 1.0 mM MgCl_2_, 10 mM HEPES, pH 7.4) supplemented with 10 mM glucose aerated for at least 30 min at 40°C with 100% O_2_, and the other end of the sac was tied using sutures. Next, the sacs were filled with KRB solution containing 10 mM glucose and incubated at 40°C for 60 min while bubbling with 100% O_2_ in a water bath. At the end of the experiment, the contents of the sacs and glass flasks were collected to analyze glucose concentrations with a Glucose Analyzer (DKK-TOA Co., Ltd., Tokyo, Japan). The contents of the sacs represented the serosal compartment, while those of the glass flask represented the mucosal compartment.

### Short-Circuit Current

The short-circuit current (*I*_sc_) experiment was performed according to previously reported methods (Shibata et al*.,* 2019). Briefly, the intact distal jejunum was excised from the C1W (*n* = 20; 149 ± 7 g BW) and C5W (*n* = 20; 2,339 ± 62 g BW) and then rinsed with modified KRB solution (4.0 mM KCl, 127 mM NaCl, 2.0 mM CaCl_2_·2H_2_O, 10 mM HEPES, pH 7.4) containing 10 mM glucose. Next, the distal jejunum was carefully removed from the muscle layer under a stereoscopic microscope (S9D; Leica Microsystems, Wetzlar, Germany), and the mucosal tissue was mounted between 2 chambers (aperture: 0.50 cm^2^) and placed in an Ussing chamber system. Both chambers were incubated with the modified buffer solution (10 mL) for 10 min at 40°C while bubbling with 100% O_2_. Both solutions were then exchanged for the modified KRB solution without glucose. Subsequently, tissues were clamped to zero potential difference and continuously recorded for changes in *I*_sc_ using a voltage-clamp amplifier (CEZ-9100, NIHON KOHDEN Co., Ltd., Tokyo, Japan) and a PowerLab/4sp performance recording unit (ADInstruments, Sydney, NSW, Australia) via Ag/AgCl electrodes and 1 M NaCl agar bridges.

After equilibration with the modified KRB solution, D-glucose (final concentration 10 mM) or D-mannitol (10 mM) solution was applied to the mucosal side. In addition, the modified KRB solution was replaced with a modified KRB solution containing 100 μM phlorizin (SGLT1 inhibitor) on the mucosal side or 100 μM ouabain (Na^+^/K^+^-ATPase inhibitor) on the serosal side. Thereafter, D-glucose (10 mM) or D-mannitol (10 mM) solution was applied to the mucosal side. Moreover, the modified KRB solution was replaced with N-methyl-D-glucamine (**NMDG**)-based Na^+^-free modified KRB solution (127 mM NMDG, 4.0 mM KCl, 2.0 mM CaCl_2_·2H_2_O, 10 mM HEPES, pH 7.4) on both sides. After stabilization of *I*_sc_, D-glucose (10 mM) was applied to the mucosal side, and 10 min later, NaCl (100 μM) or NMDG-Cl (100 μM) solution was applied. At the end of all experiments, the tissue condition was confirmed by changes in *I*_sc_ evoked by Cl^−^ secretion with carbachol (100 μM). During all experiments, the *I*_sc_ was continuously monitored as described above.

Data are expressed as the difference (*ΔI*_sc_, μA/cm^2^) before (basal *I*_sc_) and after (peak *I*_sc_) glucose, NaCl, or NMDG-Cl challenge. Voltage command pulses (10 mV, 1-s duration) were applied to the tissue at 1-min intervals to analyze tissue conductance (*G*_t_; mS/cm^2^). Based on Ohm's law, each *G*_t_ before and after glucose or mannitol (control) challenge was calculated from the mean in the following time periods: from −5 to 0 min (basal), 0 to 5 min (immediately after stimulation), and 5 to 10 min (after stimulation).

### Scanning Electron Microscopy

The distal jejunum obtained from C1W (*n* = 5) and C5W (*n* = 5) were fixed with 2.5% glutaraldehyde in PBS (pH 7.4) at 4°C, after which they were treated with 1% tannic acid solution and re-fixed with 1% osmium tetroxide solution for 1 h. The tissues were gradually dehydrated through a graded series of ethanol (50, 70, 90, 95, and 100%), and then the ethanol was replaced with t-butyl alcohol prior to performing freeze drying (Inoue and Osatake, 1988). Following coating with Pt-Pd, the specimens were observed with a scanning electron microscope (HITACHI S-4300; Hitachi High-Technologies Corporation, Tokyo, Japan). Using ImageJ software (NIH, Bethesda, MD), villus height and width and the number of villi per area (mm^2^) were analyzed (at least a total of 12 measurements in each group).

### qPCR

mRNA expression levels of each gene in the C1W (*n* = 4–9) and C5W (*n* = 4–9) were evaluated using qPCR. cDNA synthesis was performed using total RNA obtained from the distal jejunal tissues (Shibata et al., 2019). mRNA expression levels were measured by quantitative PCR on a StepOnePlus Real-Time PCR System (Thermo Fisher Scientific KK, Kanagawa, Japan) using THUNDERBIRD SYBR qPCR Mix (Toyobo Co., Ltd., Osaka, Japan). Gene expression levels were calculated from the standard curve method. Afterward, values were normalized using β-actin as the potential reference gene. The primer sets were designed using Primer 3Plus (http://www.bioinformatics.nl/cgi-bin/primer3plus/primer3plus.cgi) and are shown in Table 1.

**Table 1:** Information on the primers used for real-time PCR.

Gene	Accession #		Primer sequence (5′–3′)	Product size (bp)
*Slc5a1*	NM_001293240	F	GTCCTGGCAGTGGGAGTATG	108
		R	AAGAGTGAAGCACCGATCGG	
*Atp1a1*	NM_205521.1	F	AGAGGCAGCCAAGAAATCCC	110
		R	CCAAGGGCCTGGATCATACC	
*CLDN2*	NM_001277622.1	F	GCTGCGAGATTTCCACAACC	91
		R	GAGCAGGGAGGAGATGATGC	
*CLDN5*	NM_204201.1	F	AGGTGTCAGCCTTCATCGAC	117
		R	TGGAATCGTACACCTTGCAC	
*CLDN15*	XM_00493695.3	F	GCTGGGCTGGATCTGTTCTT	90
		R	TGCTGGCACTGTATTCCTGT	
*GJA1*	NM_204586.2	F	CTGTCTTCGAGGTGGCTTTC	109
		R	AAACAGTCCACCCTGTGAGG	
*CDH1*	NM_001039258.2	F	TGTTGAGATAAGGGGCCAAG	92
		R	TGTCCGTCAGCTTCACAAAG	
*Actb*	NM_205518.2	F	TGCGTGACATCAAGGAGAAG	111
		R	GACCATCAGGGAGTTCATAGC	

*Slc5a1*: SGLT1; *Atp1a1*: Na^+^/K^+^-ATPase alpha-1 subunit; *CLDN2*: claudin2; *CLDN5*: claudin5; *CLDN15*: claudin15; *GJA1*: connexin43; *CDH1*: cadherin1; *Actb*: β-actin.

### Statistical Analysis

All results are expressed as the mean ± standard deviation (**SD**). Differences between 2 groups were tested using a 2-sided Mann-Whitney *U* test, and those between for 4 groups were assessed using Dunn-Bonferroni post hoc tests after Kruskal-Wallis test. Statistical analysis was analyzed using SPSS 25 (SPSS Inc., Chicago, IL, USA), and statistically differences were assumed at *P* < 0.05.

## RESULTS

### Changes in Blood Glucose Levels Following Oral Glucose Administration

**Figure 1:**
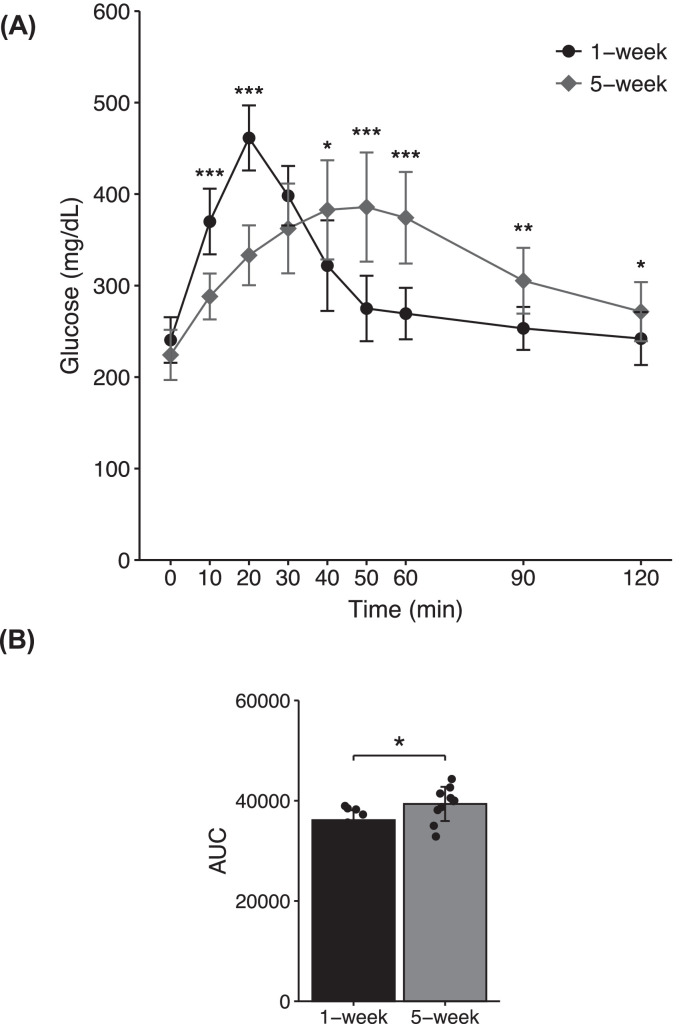
Blood glucose dynamics during oral glucose challenge. (A) Male fasted chickens at 1 wk of age (*n* = 10) and 5 wk of age (*n* = 10) received glucose solution (2 g/kg BW) by oral administration. Blood glucose concentrations were obtained at the indicated times. (B) Total area under the curve (AUC) was calculated using the trapezoidal method. Data represent the means ± SD. Statistically differences were defined as *P < 0.05, **P < 0.01, and ***P < 0.001 using 2-sided Mann-Whitney *U* test.

The C1W showed a rapid increase in blood glucose levels, reaching a peak at 20 min in response to oral glucose administration (Figure 1A). Moreover, blood glucose levels returned to baseline by 50 min. The C5W showed a gradual increase and decrease in blood glucose levels after glucose challenge; blood glucose levels reached its peak at 40 to 50 min and then returned to initial levels by 120 min. The broiler groups differed significantly in blood glucose levels at 10, 20, 40, 50, 60, 90, and 120 min after administration (*P* < 0.05). In addition, the AUC for the C5W was greater than that for the C1W (Figure 1B) (*P* = 0.035).

### Intestinal Evans Blue Transit Between the 2 Age Groups

The length of the small intestine in the C5W was greater than those in the C1W (*P* = 0.003) (Table 2). The stain ratio in the small intestine of the C5W was lower than that in the C1W (*P* = 0.01). In contrast, the tissue regions stained with Evans blue, and the migration distance of Evans blue from Meckel's diverticulum did not differ between the C1W and C5W.

**Table 2:** Comparison of small intestinal Evans blue transit between age groups.

	Small intestinal length (cm)	Stained small intestinal length (cm)	Stain ratio (%)	Distance from Meckel's diverticulum (cm)
1-wk	94 ± 11 **	67 ± 13	71 ± 10 *	6 ± 11
5-wk	148 ± 10	83 ± 12	56 ± 7	−6 ± 13

Data represent the means ± SD. Statistical significance was defined as *P < 0.05 and **P < 0.01. The location of Meckel's diverticulum (MD) was defined as zero. The stained proximal region from the MD was expressed as a negative value, whereas the stained distal region from it was expressed as a positive value.

### Intestinal Glucose Absorption Using the Everted Sac Technique

**Figure 2:**
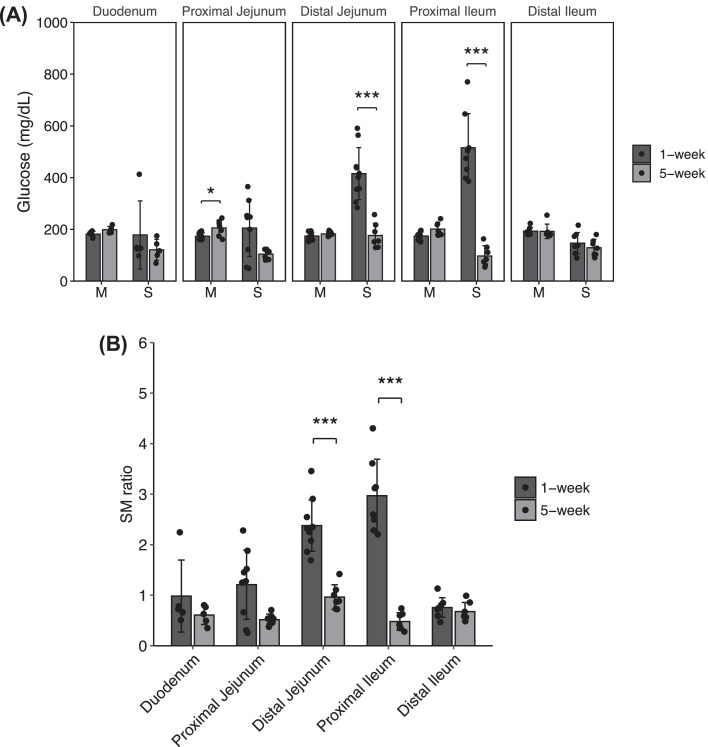
Mucosa to serosal glucose transport across everted gut sacs. (A) Everted sacs from 1-wk old (*n* = 7) and 5-wk old (*n* = 5) chickens were incubated with Krebs Ringer buffer containing D-glucose (10 mM). Glucose concentrations from mucosal and serosal solution were determined after incubation. (B) To determine the mucosal to serosal glucose transport, the glucose concentration ratio (S/M) in the serosal (S) and mucosal (M) side was estimated. Data represent the means ± SD, and 2-sided Mann-Whitney *U* tests were used to compare the glucose concentration and S/M ratios. Statistically differences were assumed at *P < 0.05 and ***P < 0.001.

The glucose levels of the mucosal compartment in the proximal jejunum were higher in the C5W than those in the C1W (*P* = 0.023). In comparison, glucose concentrations of the serosal side in the distal jejunum and proximal ileum were lower in the C5W than the C1W (*P* < 0.001) (Figure 2A). The serosal/mucosal (**SM**) glucose concentration ratio was higher at C1W than at C5W in the distal jejunum and proximal ileum (*P* < 0.001) (Figure 2B).

### Glucose-Induced Short-Circuit Current in Distal Jejunum

**Figure 3:**
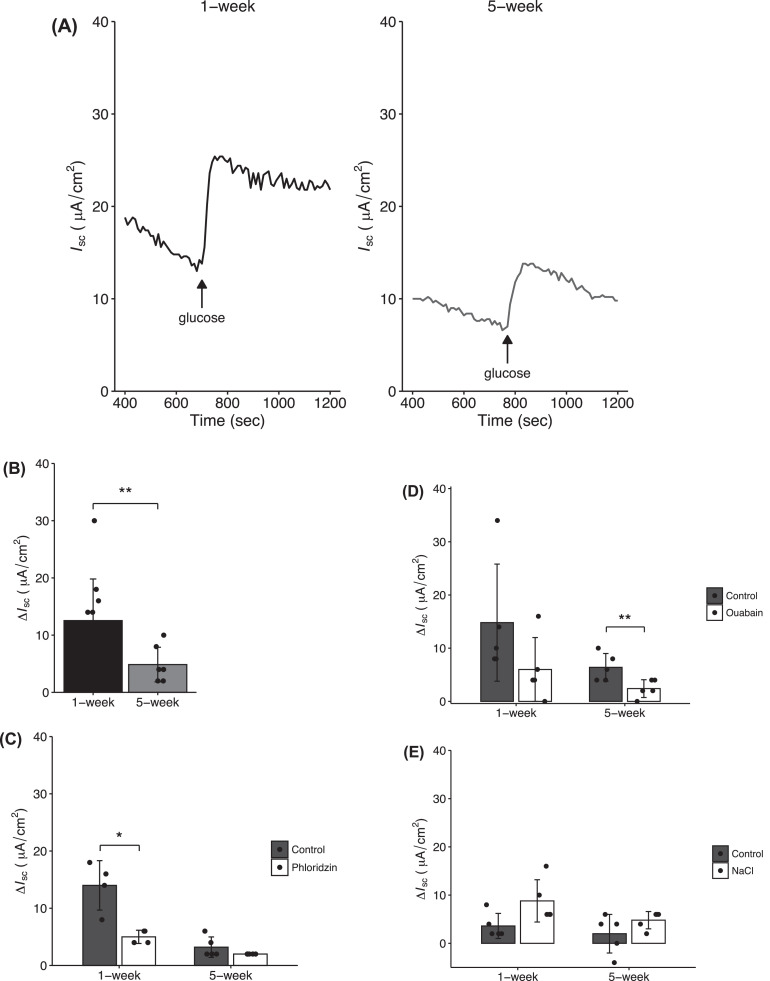
Short-circuit current (*I*_sc_) responses to glucose challenge. (A) *I*_sc_ (μA/cm^2^) recorded from the distal jejunum in response to 10 mM glucose stimulation (mucosa) in 1-wk old (*n* = 6) and 5-wk old (*n* = 8) chickens. (B) Difference between basal and maximal *I*_sc_ before and after glucose stimulation was calculated as the *ΔI*_sc_. (C) Glucose-induced *I*_sc_ in the presence of 100 μM phlorizin as SGLT1 inhibitor in mucosal side of chicks 1 wk (*n* = 5) and 5 wk (*n* = 5) of age. (D) Glucose-induced *I*_sc_ in the presence of 100 μM ouabain as Na^+^/K^+^-ATPase inhibitor on the serosal side of the 1-wk old group (*n* = 5) and 5-wk old group (*n* = 5). (E) *I*_sc_ response to NMDG-Cl (control) or 100 μM NaCl challenge using Na^+^-free modified buffer solution including glucose on both sides of the 1-wk old group (*n* = 5) and 5-wk old group (*n* = 5). Data represent the means ± SD, and 2-sided Mann-Whitney *U* tests were employed to assess the comparison of *ΔI*_sc_ values between the 2 groups (control and treatment) to evaluate the treatment effects. Statistically differences were assumed at *P < 0.05 and **P < 0.01.

The glucose-induced short-circuit current (*I*_sc_) response was elevated after stimulation with glucose in both broiler groups (Figure 3A). The *ΔI*_sc_ in the C1W was higher than that in the C5W (*P* = 0.008) (Figure 3B). The SGLT1 inhibitor phloridzin suppressed glucose-induced *ΔI*_sc_ in the C1W (*P* = 0.016), but not in the C5W (Figure 3C). The Na^+^/K^+^-ATPase inhibitor ouabain suppressed the *ΔI*_sc_ in the C5W (*P* = 0.032), but not in the C1W (*P* = 0.095) (Figure 3D). Although the addition of NaCl solution increased the *ΔI*_sc_ in the C1W, no differences between the NaCl and NMDG-Cl (NaCl-free) treatments were observed (*P* = 0.056) (Figure 3E), which was also the case in the C5W.

### Tissue Conductance in Distal Jejunum

**Figure 4:**
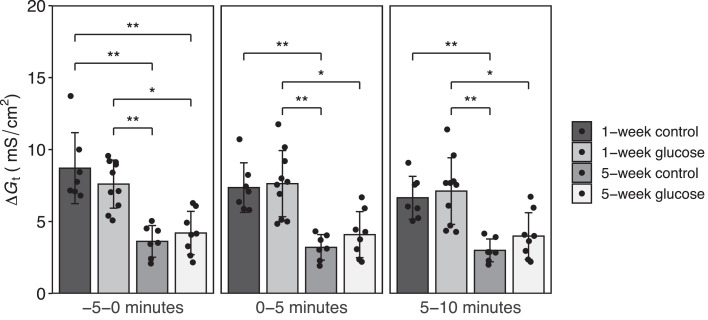
Tissue conductance in glucose challenge. Tissue conductance (*G*_t_, mS/cm^2^) was calculated as the average of each conductance before (−5 to 0 min), during (0–5 min), after (5–10 min) 10 mM D-glucose challenge in mucosal side of 1-wk old and 5-wk old chickens. Data represent the means ± SD. Dunn-Bonferroni post hoc test was used to compare the tissue conductance on 2 age and experimental groups in measurement of the glucose-stimulated *ΔI*_sc_. Statistically differences were assumed at *P < 0.05 and **P < 0.01.

In all time periods, tissue conductance in the distal jejunum did not show differences between the treatments in the same age groups (Figure 4). With or without glucose stimulation, tissue conductance in the C1W increased more than that in the C5W in all time periods (*P* < 0.05).

### Developmental Morphological Comparison of Small Intestines

**Figure 5:**
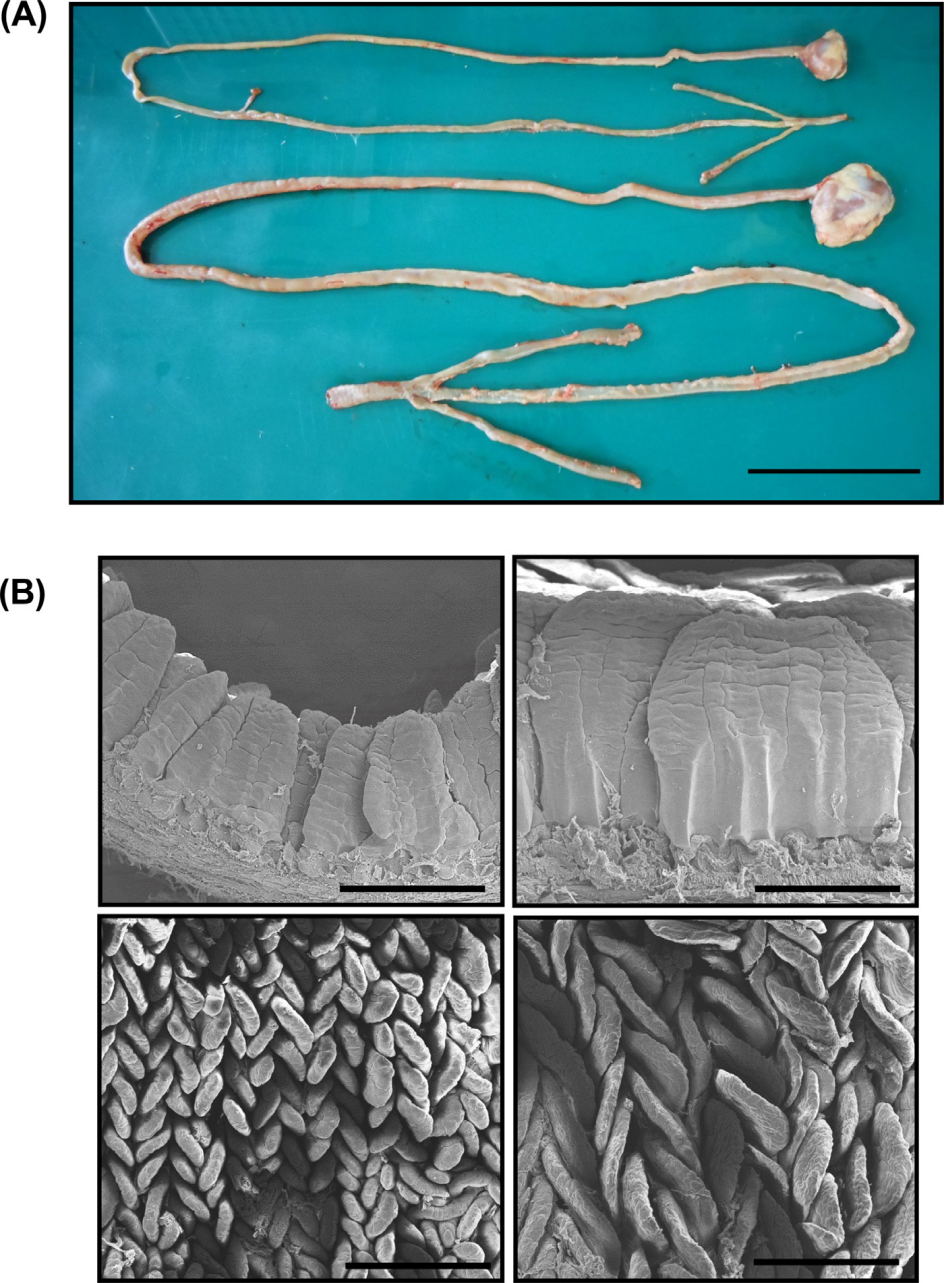
Morphological comparison of the intestine. (A) The entire intestinal length was measured from duodenum connected to the pylorus of the stomach to the end of the ileum in chicks at 1 wk (top) and 5 wk (bottom) of age, and representative images are shown. Scale bar = 10 cm. (B) Using scanning electron microscopy (SEM), the morphology of the jejunal villi in 1- (left) and 5-wk-old (right) chickens were evaluated from the side (top) and top (bottom), and representative SEM images are shown. Scale bar = 500 μm.

Small intestinal length and long and short villus diameter in the C5W were greater than those in the C1W (*P* < 0.05) (Figure 5A and B and Table 3). The number of villi per unit area (mm^2^) in the C5W was lower than that in the C1W (5-wk vs. 1-wk; 23 ± 3.8 vs. 58 ± 9.4, *P* = 0.008) (Table 3). The villi of the C1W were smaller and densely distributed than those of the C5W (Figure 5B). The dimensions of the villi were 0.21 to 0.39 mm wide and 0.32 to 0.71 mm length in the C1W, and 0.56 to 0.86 mm wide and 0.51 to 1.03 mm length in the C5W.

**Table 3:** Features of small intestinal between different age groups.

	Small intestinal length (cm)	Long diameter of villus (mm)	Short diameter of villus (mm)	The number of villus/mm^2^
1-wk	93 ± 8.1	0.49 ± 0.06	0.28 ± 0.04	58 ± 9.4
5-wk	151 ± 21.2	0.77 ± 0.09	0.72 ± 0.1	23 ± 3.8

Data represent the means ± SD. Statistical significance was defined as *P < 0.05, **P < 0.01, and ***P < 0.001.

### Gene Expression Levels in Distal Jejunum

**Figure 6:**
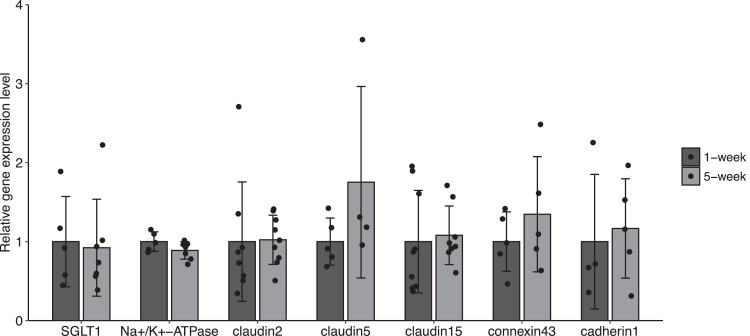
mRNA expression levels on glucose transport- and cell junction-related genes. mRNA expression levels of SGLT1 (*Slc5a1*), Na^+^/K^+^-ATPase (*Atp1a1*), claudin2 (*CLDN2*), claudin5 (*CLDN5*), claudin15 (*CLDN15*), connexin43 (*GJA1*), cadherin1 (*CDH1*) in the distal jejunum of chicks at 1 wk (*n* = 4–9) and 5 wk (*n* = 4–9) of age were analyzed using qPCR analysis. Data represent the means ± SD. Differences between groups were evaluated by 2-sided Mann-Whitney *U* test.

SGLT1 and Na^+^/K^+^-ATPase mRNA levels did not exhibit differences between the broiler groups (Figure 6). Moreover, we observed no differences in claudins 2, 5, and 15, connexin 43, and cadherin 1 between the broiler groups (Figure 6).

## DISCUSSION

Intestinal glucose absorption during growth in chickens is still unclear due to several inconsistencies. In the present study, we investigated this issue in C1W broiler chicks and C5W broiler chickens through glucose loading, Evans blue transit, and intestinal glucose absorption and permeability tests, gene expression, and morphological analyses. The dynamics and levels of blood glucose drastically differed between chicks and chickens after oral glucose challenge. In our previous study, we found that oral maltose loading alters blood glucose levels by reducing maltose-derived glucose absorption from the small intestine with growth, and this effect is independent of maltase activity in the small intestine (Shibata et al., 2019). In the present study, we performed morphological analysis and the Evans blue transit test to determine the differences in blood glucose dynamics and levels since small intestinal development affects nutrient absorption in some animal species (King et al., 2000; Buddington et al., 2001; Barfull et al., 2002; Wang et al., 2019). The C5W had significantly longer intestinal tracts and enlarged villi than those of the C1W, suggesting enhanced nutrient absorption. Furthermore, the staining ratio of the small intestine was lower in the C5W group because the intestinal length was clearly greater. The intestine of the C5W was stained up to the distal jejunum near Meckel's diverticulum, where glucose absorption is most active (Amat et al., 1996). Moreover, the stained distance from Meckel's diverticulum was no different in both broiler groups, suggesting that glucose reached the main absorption site 20 min after oral administration in both broiler groups, and other factors besides intestinal length are attributed to blood glucose levels and dynamics.

To examine intestinal glucose absorption in detail between different aged chickens, we used the everted sac and Ussing chamber techniques. Consistent with our previous results using maltose (Shibata et al., 2019), the everted sac experiment showed no increase in glucose concentration on the serosal side in the C5W. In the Ussing chamber experiment, glucose-induced *ΔI*_sc_ was lower in the C5W than the C1W. Intestinal glucose is transported into intestinal epithelial cells via SGLT1, which is primarily expressed in the apical membrane and delivers glucose into the cell using the sodium gradient created by Na^+^/K^+^-ATPase (Mueckler, 1992). The glucose is then transported into the bloodstream by facilitated diffusion via GLUT2 in the basolateral membrane. To evaluate whether this molecular mechanism was adequately functional in the 2 broiler groups, we performed the intestinal glucose absorption test under phlorizin (SGLT1 inhibitor), ouabain (Na^+^/K^+^-ATPase inhibitor), NaCl, or NMDG-Cl (Na^+^-free) incubation. Phlorizin suppressed glucose-induced *ΔI*_sc_ in the C1W but not in the C5W. Although we did not observe a significant increase in *ΔI*_sc_ of both age groups, glucose stimulation under NaCl incubation was more pronounced in the C1W (*P* = 0.056) than in the C5W (*P* = 0.22). From these results, we expected intestinal SGLT1 expression levels to vary between broiler groups, but SGLT1 mRNA expression levels did not differ, consistent with our previous study (Shibata et al., 2019). In agreement with our result, Miyamoto et al. (1992) observed that SGLT1 mRNA levels did not change during development in rats. In contrast, SGLT1 density in the jejunal BBM declined in 5-wk-old layer chickens (Barfull et al., 2002). Furthermore, SGLT1 mRNA expression levels were linearly upregulated in broiler chickens posthatch (Sklan et al., 2003; Gilbert et al., 2007). Although these reports are conflicting, the present results suggest that reduced SGLT1 sensitivity may trigger the decrease in intestinal glucose absorption, independent of SGLT1 mRNA expression levels. Moreover, we cannot rule out the effects of changes in SGLT1 protein levels since they were not measured in the present study. Meanwhile, ouabain suppressed glucose-induced *ΔI*_sc_ in both broiler groups, but not in the C1W (*P* = 0.095). Since Na^+^/K^+^-ATPase activity in the colon exhibits an age-correlated increase in rats (Finkel et al., 1985), it is possible that Na^+^/K^+^-ATPase of the C5W chickens are more functional than of the C1W chicks.

In the present study, membrane conductance was lower in the C5W than the C1W, indicating that paracellular ion permeability decreased in the C5W. Substance permeability via the paracellular pathway in the intestine of neonates is higher than that in adult animals (Wisser and Horster, 1978). Notably, deficiency of tight junction proteins claudins 2 and 15 reduces Na^+^ supply from the submucosa to the intestinal lumen, resulting in diminished Na^+^-dependent nutrient absorption, including that of glucose and amino acids (Tamura et al., 2011; Wada et al., 2013). More recently, Nakayama et al. (2020) provided direct evidence that Na^+^ taken up from the lumen into the submucosa diffuses again into the lumen through a paracellular pathway to facilitate nutrient absorption via Na^+^-dependent cotransporters, and they also revealed that claudin 15 is necessary for the Na^+^ recycling system. Additionally, claudin 15-deficient mice exhibit megaintestine by increasing intestinal length and villus size in the upper small intestine (Tamura et al., 2008). Therefore, in the present study, we expected claudins 15 and 2 to be involved in the age-related differences in intestinal glucose absorption. However, mRNA expression levels of cell junction-related genes, including claudins 15 and 2, did not show differences between the C1W and C5W. Taken together, ion permeability, independent of claudins 15 and 2, may partially contribute to the age-related differences in intestinal glucose absorption in broiler chickens. Other claudins may be associated with recycling Na^+^ from the submucosa into the lumen. Moreover, we found changes in intestinal ion permeability with growth but did not investigate Na^+^ permeability. In the future, more detailed regulatory mechanisms of growth-associated intestinal glucose absorption will be revealed by examining Na^+^ permeability in the intestine.

In addition to these regulators, we must consider glucose absorption throughout the entire intestine. Since the everted sac and Ussing chamber experiments depend on a portion of the intestinal tract, the results do not necessarily represent the function of the entire intestinal tract. In fact, although intestinal glucose absorption per unit area decreased with growth in the present study, the AUC of the glucose infusion test suggests that glucose absorption throughout the intestinal tract is greater at 5 wk of age than that at 1 wk of age. These findings imply that local glucose absorption declines with growth, but glucose absorption in the entire intestine increases with morphological gut development. Interestingly, we previously observed that local amino acid absorption in the intestine is higher in C5W than in C1W, which is inconsistent with that observed for glucose (Shibata et al., 2020). Therefore, the factors discussed above may be actively involved in intestinal glucose absorption in addition to morphological differences between the broiler groups.

In summary, blood glucose levels and their fluctuation after oral glucose challenge vary greatly with age, that is, from 1 to 5 wk, in meat-type chickens, and decreased SGLT1 sensitivity leads to a corresponding decrease in intestinal glucose transport at 5 wk of age. In addition, tissue conductance, which indicates the integrity of tight junctions that regulate ion permeability, is diminished in the chickens at 5 wk of age compared with that in the chicks at 1 wk of age. Moreover, in chickens at 5 wk of age, the intestinal tract is developed, jejunal villi show hypertrophy-like signs, and glucose absorption throughout the entire intestine is greater than that in 1-wk-old chicks. However, the factors underlying the decline in intestinal glucose transport with growth observed in this experiment were not clarified. Because the gut environment (gut microbiota and metabolite levels) varies dramatically with growth, these underlying factors may contribute to this decline. In addition, further research on laying hens and other bird species would help to better understand the specificity of this phenomenon. The present study provides insights for characterizing intestinal glucose absorption in chicken, which is a unique bird among birds, and will hopefully lead to the development of novel feeds that consider this characteristic.
